# Examining influencer compliance with advertising regulations in branded vaping content on Instagram

**DOI:** 10.3389/fpubh.2022.1001115

**Published:** 2023-01-09

**Authors:** Nathan A. Silver, Adrian Bertrand, Padmini Kucherlapaty, Barbara A. Schillo

**Affiliations:** Truth Initiative, Washington, DC, United States

**Keywords:** advertising, promotion, tobacco industry, social media, Instagram

## Abstract

**Background:**

Youth and young adults are exposed to vaping advertisements on social media sites, despite regulations and guidelines intended to reduce the prevalence of such content on these platforms. This research uses replicable criteria to identify vaping influencers who have worked with vaping brands to promote vaping products on Instagram and documents the extent to which posts by these users comply with existing advertising regulations.

**Methodology:**

We conducted three google searches collecting eight different vaping influencer lists, with a total of 575 unique influencers. We limited our sample to public accounts with 100,000 followers or more (*n* = 54). An initial sample of 360 Instagram posts was used to identify an analytic sample of 262 vape-related posts from 2021. We conducted a conceptual content analysis to first identify unambiguous vaping advertisements (branded content), and then code ads for compliance with existing regulations.

**Results:**

On average, the 54 Instagram accounts had 265,851.9 followers (sd = 383,349.8) and 4,158 posts (sd = 7,302.1). Most posts featured vaping products 239 (91.2%), with 186 (76.2%) posts being unambiguously branded vape advertisements and 31 (14.3%) even including purchase links in the post itself. However, one post complied with FTC disclosure guidelines. Although 50 (20.9%) had warning labels, only 8 (15.1%) were fully compliant with FDA warning label guidelines.

**Discussion:**

Findings demonstrate minimal compliance with existing regulations among influencers known to have financial relationships with vaping brands. Most influencer posts are unambiguous, branded, vaping advertisements. Implications for barriers to regulating influencer content and the need for greater enforcement resources are discussed.

## 1. Introduction

Youth and young adults are often exposed to tobacco advertising through social media use. (1–3) As of 2021, an estimated 84% of young adults reported using at least one social media site (4, 5). Moreover, 53% of American youth report past 30-day exposure to tobacco advertisements on social media sites (1). Instagram, a visual social media platform most popular among youth and young adults (6), is particularly problematic, as an increase in vaping-related content (7) has created a platform where users are far more likely to be exposed to visually appealing vape advertisements than tobacco control and educational content (8). Exposure to vaping content on social media sites is associated with both lower risk perceptions as well as increased likelihood of initiation and habitual use of vaping products (9, 10). Youth vaping remains a prevalent public health problem with documented habitual use as early as middle school and an estimated 14.1% of high school students (11) and 9.7% of young adults 18–24 reporting current use (12). In light of the clear pathway between exposure to vaping content on social media and use and progression of vaping behavior, updated marketing policies that extend to social media platforms like Instagram are needed.

The stated aim of tobacco marketing restrictions in the Master Settlement Agreement (MSA) is “prohibiting tobacco companies from taking any action to target youth in the advertising, promotion, or marketing of tobacco products” (13). Youth-targeted ad characteristics like cartoons are strictly prohibited in advertisements promoting cigarettes and smokeless tobacco products (14). Moreover, “youth-targeting” was also conceptualized as the placement of ads where young people are likely to be exposed. As a result, promotional materials are not allowed near schools, at sporting events, or in youth-oriented magazines. Furthermore, cigarettes and smokeless tobacco cannot be promoted on broadcast media given the probability of youth exposure (14). Although a clear goal of the MSA was to keep tobacco ads off of platforms where youth exposure was likely, the MSA does not apply to newer products, like vaping, or newer mediums, like social media. Thus, there is a need for updated restrictions on advertising that are consistent with the current tobacco product and media landscape.

In the years following the deeming of e-cigarettes as tobacco products to be regulated by The U.S. Food and Drug Administration (FDA) Center for Tobacco Products (CTP), FDA CTP has issued guidance as to what constitutes youth appealing advertising, specifically highlighting the way “Marketers seek to create “brand ambassadors,” [i.e., social media influencers] who promote the product in the context of their online communications” (15). However, the ambiguous space between private citizens and hired brand representatives occupied by social media influencers has made effective regulation elusive. Vaping companies often contract with influencers who are paid for their ability to target a niche audience (16) of potential buyers (17); and have become a vehicle for promoting products on social media, especially Instagram (18). Because influencers can also share organic, unpaid content, it can be difficult to identify which posts are subject to commercial regulation (18).

Instagram posts are potentially subject to a hodgepodge of existing regulations. The U.S Food and Drug Administration (FDA) prohibits unsubstantiated claims about health benefits or modified risk of vaping (19), and requires a nicotine health warning label to occupy at least 20% of an advertisement (20, 21). The Federal Trade Commission (FTC) issued guidelines for disclosing relationships between social media influencers and brands (22). Finally, Instagram's branded content policies explicitly prohibit promotion of “tobacco products, vaporizers, electronic cigarettes, or any other products that simulate smoking” (23). However, previous research shows low compliance with FDA nicotine health warning requirements (24), low compliance with FTC disclosure guidelines among social media influencers (25), and limited impact of such restrictions on the volume of branded content still prevalent on Instagram (26). At the time data was collected for this study, hashtags such as #vape (31 million posts), #vapelife (17 million posts), and #vapecommunity (11.9 million posts) highlight the prevalence of vape-related content, while #vapestore (2.49 million posts) clearly indicates that a substantial portion of such posts are commercial (27).

The current research is based on the assumption that limited enforcement of existing regulations observed by previous research is the result of ambiguity regarding commercial vs. organic posts. Like previous research, we examine Instagram posts for compliance with existing regulatory guidelines. However, we go one step further in adding “explicitly branded content” by “known hirable entities” as criteria for inclusion. To inform the development of a policy framework for regulating advertising on Instagram, we take the perspective of a vaping brand seeking to hire social media influencers to promote vaping products on Instagram. We first identify a sample of known vaping influencers (i.e., hirable content creators easily identified *via* web search), capture a sample of their most recent content, identify branded vape content from within their most recent content We thus propose two research questions:

RQ1: What percentage of the most recent posts by known vaping influencers are unambiguously ads (i.e., promote branded vaping content)?
RQ2: How often are unambiguous vaping ads by vaping influencers in violation of existing regulations?

## 2. Methods

### 2.1. Procedure

A replicable protocol was established to systematically sample posts from known vaping influencers on Instagram who are available for hire. We then conducted a conceptual content analysis with two trained coders to identify posts that met our proposed criteria for unambiguous vape ads (e.g., promoted branded vaping products for sale) and identify their compliance with existing regulations.

### 2.2. Influencers and content sampling

**Figure 1:**
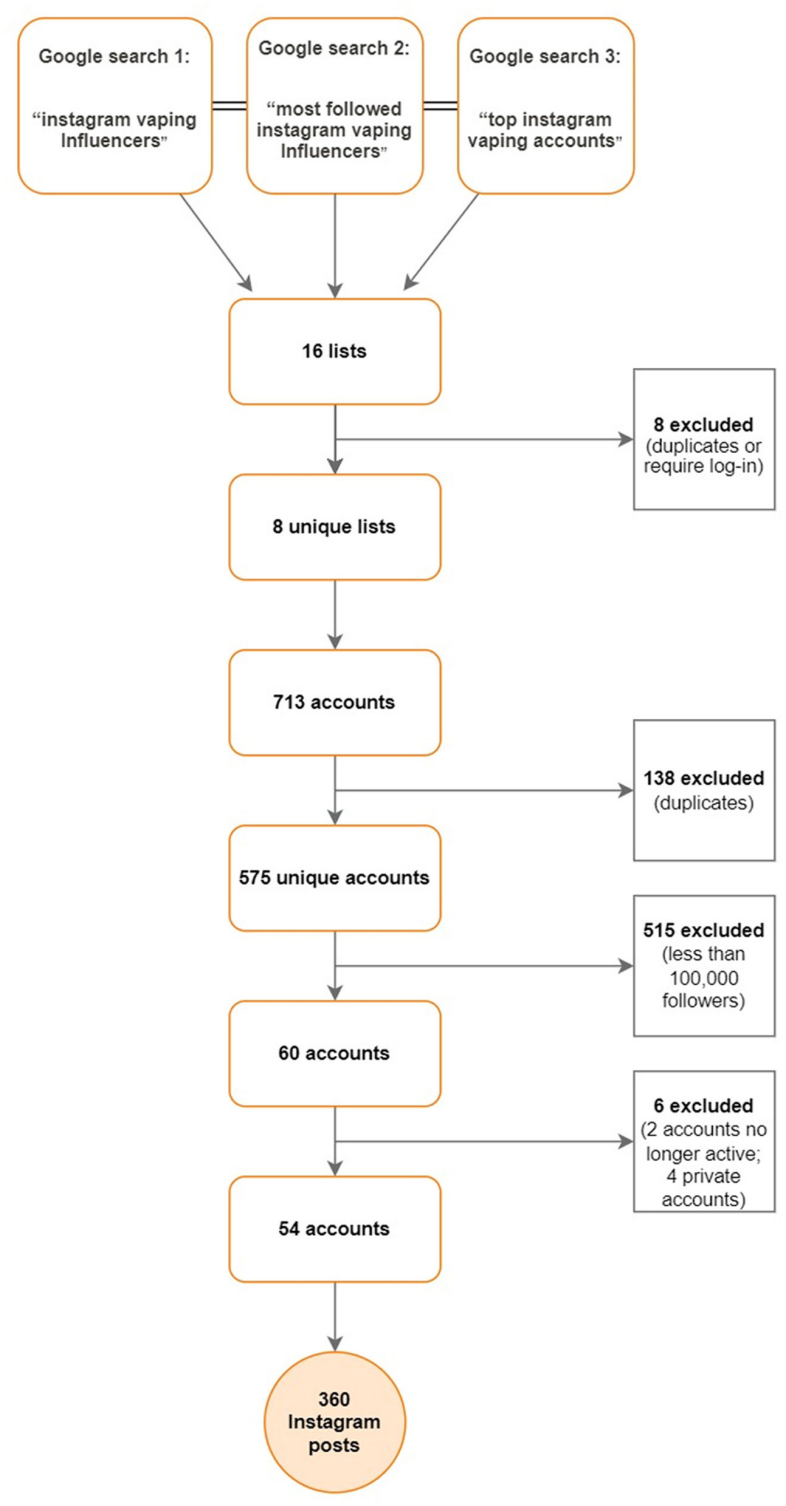
Flow chart of key search terms, lists, and post selection.

We sought to mimic the process a brand might go through in finding an influencer to hire to promote a vaping product. We developed an online google search protocol to identify websites listing vaping influencers active on Instagram using an established definition of an influencer as “third-party actors who have established a significant number of relevant relationships to influence on organizational stakeholders through content production, content distribution, interaction, and personal appearance on the social web” (28). These lists were compiled by bloggers, sites advertising influencers for hire or Instagram users outside of the platform and required no log-in to access them and provided links to each influencer's account. Our Google searches provided an initial sampling frame of 575 unique accounts, with between 1,042 and 2.6 million followers each. From those, we focused on the top 60 accounts with 100,000 followers or more. Two accounts were no longer active, and four were private, giving us a user sample of *n* = 54 unique vaping influencers. To sample the influencers' most recent activity, starting in October 2021, we downloaded a maximum of ten of the most recent posts from 2021. Several users had <10 posts from 2021, yielding an initial sample of *n* = 360 posts made by vaping influencers unambiguously identified as available for hire to promote vaping brands on Instagram. The full search protocol is documented in Figure 1.

### 2.3. Codebook development

Our codebook and content analysis followed best practices established in *The Content Analysis Guidebook* (29). It is recommended that at least 10% of the sample be withheld to establish reliability, and that researchers and coders set multiple coding practices and meet frequently to discuss and resolve discrepancies. Given that we coded for concrete features that can be discreetly identified as present or absent (e.g., warning labels, disclosures etc.) rather than abstract features that are more subject to interpretation (e.g., ad themes or emotional appeals), we set a higher threshold for reliability of Krippendorff's alpha >0.8 and trained two research assistants to code the same reliability sample of *n* = 66 posts. After reliability was established, the remaining posts were divided equally between the two coders. Coders first identified vape posts which were then coded for unambiguous ad characteristics (e.g., branded content), FTC brand relationship disclosure violations, and FDA claims or warning label violations. Table 1 presents all coded features, a summary of criteria used to identify coded features, and the resulting Krippendorff's alpha values.

**Table 1:** Krippendorff's alpha accessing reliability of coded post characteristics with summary of criteria.

**Category**	**Post characteristic**	**Summary of criteria**	**α**
Vape ad	Vaping product	Presence of vaping products such as devices or device parts or e-liquids	1
	Branded	Presence of a product brand is clearly visible within the image	0.90
Instagram violation	Company	Post was by a specific company-owned account	0.96
FTC violation	Links to purchase	Post included link or redirection (link in bio) about where to buy an advertised product	0.95
	Review	Presence of a critical assessment or evaluation of the product featured on the image	0.94
	Disclosure	The presence of a statement indicating a financial relationship between the influencer and the product or brand in the post, including money, gifts, or other perks of monetary value	0.91
FDA violation	Modified risk/Harm reduction	Presence of a statement indicating the product featured in the image as being a modified risk tobacco product	0.97
	Warning label	Presence of health warning labels	0.97
	Warning Label and Image	Presence of a warning label across the post and covers 20% of the image	1
	Cartoon image	Presence of a cartoon character in the image	0.96
	Flavor visible	Presence of a flavor clearly visible within the image	0.82

### 2.4. Identifying relevant content

Not all posts by vaping influencers were related to vaping. We developed strict rules to determine whether a post was a vaping post or not. Our two coders identified the presence of at least one of the following key elements in the image: a vape device or parts including e-juice, coils, mouthpieces etc., 258 (98.47%), kalpha = 1, vaping behavior such as a vape cloud, 85 (32.44%), kalpha = 1, or a person, 80 (30.53%), kalpha = 1, or group of people, 2 (0.76%), kalpha = 1, actively drawing (inhaling from a vape). Ambiguous images (e.g., a cloud potentially though not clearly from vapor) were resolved using explicit cues in the text of the post (e.g., a vaping brand or hashtag that linked the cloud to vaping). We focused our analyses on *n* = 262 unambiguous vaping posts from *n* = 54 vaping influencers on Instagram.

## 3. Results

Information about each influencer was pulled directly from the bio section of their Instagram account. On average, the 54 vape influencer accounts had 265,851.9 followers (sd = 383,349.8) and 4,158 posts (sd = 7,302.1). The influencer accounts were not limited to individual people *n* = 28 (51.8%), but also included brands, *n* = 15 (27.8%), and other commercial entities such as retailers or shops, *n* = 17 (31.5%), both of which are violations of Instagram's internal policy. Influencer bios included links to vaping stores *n* = 17 (31.5%). The accounts also had hashtags in their bios *n* = 17 (31.5%) including vape-specific hashtags (e.g., #vaping, #vapelife).

At the post level, nearly all posts featured vaping products, *n* = 239 (91.2%). Hashtags intended to reach online vaping community members (e.g., #vapelife, #vapefam, #vapeon) were frequent throughout the sample. Despite 186 posts (76.2%) being unambiguously branded, only 1 post complied with FTC requirements of disclosing brand relationships. Moreover, 31 (14.3%) posts provided links to purchase the advertised product, signifying but not disclosing a paid relationship between influencer and brand. Violations of FDA's rules regarding unauthorized claims were rare (one post highlighted modified risk). However, only 50 posts (20.9%) had any warning label, with 8 (15.1%) of those being fully compliant with 20% of the image covered by a warning label. Finally, three posts used cartoon imagery in opposition to the FDA's warning about use of youth appealing advertisement.

## 4. Discussion

This research adds to the growing body of evidence highlighting the importance of addressing tobacco promotion on Instagram (3, 24, 26, 30–32). Although social media and the use of influencers pose unique challenges to regulators (18), our findings suggest that a significant amount of content includes egregious violations of already established rules. Nearly half of the influencer accounts we identified were vaping brands or retailers, many of whom use hashtags such as #vapelife, #vaping, and #vapefam among others in their posts to disseminate such content among 10s of millions of ostensibly organic posts, all in clear violation of Instagram's stated policy. Moreover, with more than 90% of posts featuring specific products and more than 75% of posts including obvious branding, most of these posts were unambiguous vaping ads—explicit promotion of branded vaping products. Only eight were compliant with FDA guidelines and one was in compliance with FTC guidelines.

The most important takeaway form this research is that FTC disclosure guidelines for influencers to disclose brand relationships are insufficient. Our design identifies influencers who actively seek and maintain relationships with brands, post branded content that unambiguously promotes specific vaping products, and are for the most part, not in compliance with FDA, FTC, or Instagram advertising guidelines. In conjunction with previous research identifying content promoting vaping on Instagram (26, 33), it is clear that greater resource allocation toward enforcement of existing regulations is needed.

Our findings also highlight the limitations of regulating influencer-based advertising at the level of the brand-influencer relationship. Like previous research, we show clear collaboration between brands and influencers to promote specific vaping products (24, 25). Conceptually, promotion of a specific brand or line of products is an ad. However, legally, in the absence of a disclosed financial relationship, such posts may be interpreted as organic and thus not subject to advertising restrictions. Regulating broadcast or print media at the level of the financial relationship between medium and brand was effective because billboards, ad space on TV, radio, magazines, or sports stadiums all necessarily implied a financial relationship wherein brands purchased ad space and were thus subject to advertising regulations. Posting on Instagram about a favorite product does not necessarily entail a financial relationship. In fact, there is undoubtedly a disincentive to disclose otherwise obvious relationships, as doing so would subject posts to regulatory scrutiny to which organic posts are exempt. Regulation of vaping promotion on Instagram likely requires a legal and conceptual definition of an ad that can be applied at the level of the post, as our analysis suggests the status quo is ineffective.

The generalizability of these findings is limited by our sampling method. We focus on influencers whose financial relationships to vape brands are easily identifiable through a systematic web search. As a result, our sample of influencers is likely not representative of the far broader population of vape influencers employed by vaping brands and the tobacco industry. Moreover, by focusing on the last 10 posts rather than a random sample of each users' post history we provide more of a “snapshot in time” rather than a generalizable accounting of the percentage of influencer posts that are in violation of extant guidelines. Nevertheless, this study provides strong evidence that vaping ads comprise a significant portion of vaping content, but still appear to be exempt from regulatory enforcement.

### 4.1. Conclusion

There are inevitable challenges to regulating content on a platform like Instagram where organic users and users with commercial interests alike contribute to a seemingly endless onslaught of content that runs the gamut from personal artistic expression to branded product advertisements. Clear, unambiguous guidelines are needed to differentiate commercial content from organic content. The aim of a lot of vaping content on Instagram is not ambiguous including clearly branded and commercial-oriented promotion of specific vaping products. This study adds to the mounting pile of evidence highlighting the need for better enforcement of existing guidelines while also highlighting the potential need for better defined parameters for identifying commercial content subject to regulatory enforcement.

## Data availability statement

The datasets used in this study are publicly available on Instagram. The weblinks for all content used has been collated in the supplementary material. A copy of the coding instrument used can be made available upon request to the corresponding authors.

## Author contributions

This study was conceptualized by NS and AB. The codebook was created by AB with input and supervision by NS and BS. PK supervised coding and conducted our analysis. NS, AB, PK, and BS contributed to the writing of the manuscript. All authors contributed to the article and approved the submitted version.
